# Apolipoprotein A-I glycation by Glucose and Reactive Aldehydes Alters Phospholipid Affinity but Not Cholesterol Export from Lipid-Laden Macrophages

**DOI:** 10.1371/journal.pone.0065430

**Published:** 2013-05-31

**Authors:** Bronwyn E. Brown, Estelle Nobecourt, Jingmin Zeng, Alicia J. Jenkins, Kerry-Anne Rye, Michael J. Davies

**Affiliations:** 1 The Heart Research Institute, Sydney, New South Wales, Australia; 2 Department of Medicine (St Vincent's), The University of Melbourne, Melbourne, Victoria, Australia; 3 Faculty of Medicine, University of Sydney, Sydney, New South Wales, Australia; Innsbruck Medical University, Austria

## Abstract

Increased protein glycation in people with diabetes may promote atherosclerosis. This study examined the effects of non-enzymatic glycation on the association of lipid-free apolipoproteinA-I (apoA-I) with phospholipid, and cholesterol efflux from lipid-loaded macrophages to lipid-free and lipid-associated apoA-I. Glycation of lipid-free apoA-I by methylglyoxal and glycolaldehyde resulted in Arg, Lys and Trp loss, advanced glycation end-product formation and protein cross-linking. The association of apoA-I glycated by glucose, methylglyoxal or glycolaldehyde with phospholipid multilamellar vesicles was impaired in a glycating agent dose-dependent manner, with exposure of apoA-I to both 30 mM glucose (42% decrease in k_slow_) and 3 mM glycolaldehyde (50% decrease in k_fast_, 60% decrease in k_slow_) resulting is significantly reduced affinity. Cholesterol efflux to control or glycated lipid-free apoA-I, or discoidal reconstituted HDL containing glycated apoA-I (drHDL), was examined using cholesterol-loaded murine (J774A.1) macrophages treated to increase expression of ATP binding cassette transporters A1 (ABCA1) or G1 (ABCG1). Cholesterol efflux from J774A.1 macrophages to glycated lipid-free apoA-I via ABCA1 or glycated drHDL via an ABCG1-dependent mechanism was unaltered, as was efflux to minimally modified apoA-I from people with Type 1 diabetes, or controls. Changes to protein structure and function were prevented by the reactive carbonyl scavenger aminoguanidine. Overall these studies demonstrate that glycation of lipid-free apoA-I, particularly late glycation, modifies its structure, its capacity to bind phospholipids and but not ABCA1- or ABCG1-dependent cholesterol efflux from macrophages.

## Introduction

People with diabetes have a greater risk of mortality from cardiovascular disease (CVD) [1] and those with poor glycaemic control or renal damage, manifest multiple pro-atherogenic risk factors including abnormalities in lipoprotein composition, subclass distribution and metabolism [2]. These factors do not however fully explain the increased CVD risk. Intensive management of Type 1 diabetes reduces CVD events, with most of the decreased risk related to lower HbA_1c_ levels [3] implicating hyperglycaemia as a major factor [4].

Hyperglycaemia results in increased non-enzymatic reaction of sugars with proteins. This involves three components: non-oxidative addition of sugar to the protein (glycation), and auto-oxidation of both free and protein-bound sugars (glycoxidation or late glycation) [5]. Glucose oxidation, enhanced glucose metabolism (via triosephosphates [6]) and glycation, yields aldehydes including glyoxal, methylglyoxal and glycolaldehyde [5]. These species are usually elevated in people with diabetes and correlate positively with disease duration and worse glycaemic control [6], [7]. These aldehydes react more rapidly than glucose, via addition to lysine (Lys), alpha-amino, arginine (Arg), histidine (His), tryptophan (Trp), and cysteine (Cys) residues of proteins; these reactions yield, ultimately, ‘late glycation’ or advanced glycation end-products (AGEs) [5]. A major AGE, N^ε^-carboxymethyllysine (CML) is elevated in plasma and atherosclerotic lesions from people with diabetes [8]. Oxidised or heavily glycated low-density lipoproteins (LDL) are recognised by macrophage scavenger receptors resulting in the formation of lipid-laden (foam) cells [9], [10] whereas native, or only mildly-modified, LDL is internalised by classical LDL receptors.

HDL, and its main protein component, apolipoprotein A-I (apoA-I), act as cholesterol acceptors, resulting in net cholesterol efflux from macrophages in atherosclerotic lesions [11]–[13]. Efflux to lipid-poor apoA-I occurs via binding to ATP-binding cassette transporter A-1 (ABCA1) and subsequent lipidation by cellular phospholipids and cholesterol, forming discoidal HDL [11]. Plasma factors, including lecithin:cholesterol acyltransferase (LCAT) remodel discoidal HDL to form spherical HDL, with excess cholesterol cleared by the liver [12]. Efflux to discoidal or spherical HDL particles occurs via ABCG1 [13]. ABCA1 and ABCG1 expression are regulated by liver X (LXR) and retinoid X receptors (RXR), cellular cholesterol levels, and oxysterols. ABCA1 transcription is stimulated by cAMP in mouse macrophages [11], [13]. Cholesterol efflux may also occur via diffusion in a process that is dependent on scavenger receptor BI (SR-BI) and is affected by the concentration gradient, phospholipids, and external acceptors, as the SR-BI pathway is bi-directional [11].

AGEs are elevated in apoA-I from people with diabetes, and also in apoA-I modified *in vitro* by incubation with methylglyoxal [14]. Glycation of apoA-I by methylglyoxal affects the functional properties of HDL, such as its ability to activate LCAT [15]. ApoA-I may also be glycated *in vitro* by fructose or artificial sweeteners with this reported to enhance cell senescence and uptake of modified LDL [16], [17]. Pretreatment of cells with glycolaldehyde has also been shown to impair cholesterol efflux to apoA-I [18] and HDL [19] via modification of ABCA1 and ABCG1 expression. Studies on the effects of HDL glycation/glycoxidation on cholesterol efflux have yielded mixed data, with both impairment and no effect reported [20]–[23]. We therefore examined the hypothesis that glycation of apoA-I by reactive aldehydes would modulate phospholipid affinity and efflux of cholesterol from lipid-loaded cells. This has been investigated using homogenous and well-characterised species: lipid-free apoA-I and discoidal reconstituted HDL (drHDL, which contains phosphatidylcholine complexed with apoA-I), as well as lipid-free apoA-I from people with Type 1 diabetes and normal controls. These studies show that glycation of apoA-I occurs readily with reactive aldehydes, and less rapidly with glucose, and that this results in modification of specific protein side-chains as well as cross-linking. These changes modulate phospholipid binding but not cholesterol efflux from lipid-laden macrophage cells.

## Materials and Methods

### Ethics statement

Collection of blood for LDL isolation, from healthy volunteers, was approved by the Sydney South West Area Health Service (New South Wales, Australia); protocol numbers X09-0013 and HREC/09/RPAH/19. The clinical protocol for the collection of blood from people with diabetes and matched controls for apoA-I isolation was approved by the St Vincent's Hospital Ethics Committee (Melbourne, Victoria, Australia). All participants gave written informed consent.

### Reagents

Reagents were from Sigma-Aldrich (St Louis, USA) except for pre-cast gels and Chelex-100 resin (Bio-Rad, Australia), glucose (Merck, Australia), N^ε^-carboxymethyllysine (CML) (TRC, Canada), and 1-palmitoyl-2-oleoyl phosphatidylcholine (POPC) and 1,2-dimyristoyl-*sn*-glycero-3-phosphatidylcholine (DMPC) (Avanti Polar Lipids, Alabaster, Alabama, USA). Solutions were prepared using Nanopure water (Millipore-Waters, Australia) pre-treated with washed Chelex-100 resin to remove metal ions, with the exception of tissue culture reagents where Baxter sterile water (Old Toongabbie, Australia) or PBS were used.

### Subjects

People with well-defined (American Diabetes Association guidelines) Type 1 diabetes (n = 12) without vascular complications, and who were not on any medication other than insulin, were recruited. Apparently healthy normolipidemic, non-diabetic, age and BMI-matched controls on no medication (n = 10) were also recruited (Table 1). Plasma was isolated (2000 *g*, 15 min, 4°C) from 80 ml of blood taken into EDTA-Na_2_.

**Table 1:** Characterisation of Type 1 diabetes and control populations.

	Type 1 diabetes	Control subjects
Age (years)	32±8	34±8
Sex (M/F)	6/6	6/4
N	12	10
BMI (kg/m^2^)	24.7±2.1	24.1±2.2
HbA_1c_ (%)	7.9±1.2**	5.1±0.4
Fasting blood glucose (mM)	13.4±4.1**	5.1±0.5
Urinary albumin (µg/min)	15.1±8.3	9.8±5.6
HDL-C (mM)	1.4±0.3	1.4±0.3
Total cholesterol (mM)	4.4±0.6**	5.6±0.8
Triglyceride (mM)	0.9±0.3*	1.5±0.9

* *p*<0.05,

** *p*<0.001 compared to control population.

### Lipoprotein preparation, modification and characterisation

LDL (1.019<d<1.06 g/ml) were isolated and acetylated as previously [9]. Modification was confirmed by relative electrophoretic mobility (REM) on agarose gels [9]. HDL (1.063<d<1.21 g/ml) for *in vitro* experiments were isolated from pooled autologously donated human plasma (Gribbles Pathology, South Australia, Australia). Further HDL were isolated from people with Type 1 diabetes and normal controls. ApoA-I was isolated, and discoidal reconstituted HDL (drHDL) containing POPC and apoA-I (initial molar ratio 100∶1, final molar ratio 90–99∶1 molar ratio, determined from analysis of the particles [24]) were prepared as previously described [24]. Samples were dialysed against PBS before use.

Lipid-free apoA-I and drHDL (1 mg apoA-I protein/ml) were glycated with the concentrations of glucose, methylglyoxal or glycolaldehyde (all from Sigma-Aldrich, St Louis, USA; catalogue numbers G5767, M0252 and G6805 respectively) stated in the text in PBS at 37°C for 24 h, in sealed tubes flushed with N_2_ gas. Unreacted reagents were removed by dialysis against PBS.

### Characterisation of glycated apoA-I

Arg, Lys and Trp modification was assessed fluorometrically [25]. Protein cross-linking was determined by SDS-PAGE [9]. CML was quantified by HPLC using fluorescence detection. ApoA-I (≥400 µg protein) was precipitated using trichloroacetic acid (200 µl, 50% w/v), centrifuged (4300 *g*, 2 min), washed (2×500 µl ice-cold acetone), re-pelleted by centrifugation (7000 *g*, 2 min) and dried by vacuum centrifugation. Samples were hydrolysed, derivatised using *o*-phthaldialdehyde, and subjected to HPLC with fluorescence detection [26]. Samples were separated (flow rate 1 ml/min) using a gradient of 85% buffer A (96% 50 mM sodium acetate, pH 6.5 and 4% methanol; v/v) and 15% buffer B (100% methanol) for 35 min; 15–90% buffer B over 5 min; 90% buffer B for 2 min; 90-15% buffer B over 5 min; and re-equilibration at 15% buffer B for 8 min with buffers passing through an inline Shimadzu DGU-14A degassing unit. Identities of peaks were confirmed by spiking with authentic materials. Peak areas were converted to absolute levels using standard curves.

### Phospholipid association assay

DMPC was dissolved in a 2∶1 (v/v) chloroform/methanol solution, and dried under nitrogen, before being redissolved in TBS (Tris-buffered saline) containing 8.5% KBr, 0.01% EDTA and 0.1% NaN_3_, at a final concentration of 0.5 mg/ml to give a turbid solution of multilamellar vesicles (MLV) [27]. MLV and apoA-I (native or modified, 0.5 mg/ml in TBS) were preincubated individually at 24°C before mixing (2.5∶1 w/w) in a quartz cuvette. Samples were read at 325 nm within 15 s at 24°C using a UV-VIS spectrophotometer (UV-2550 with a TCC-240A temperature-controlled cell holder; Shimadzu, Kyoto, Japan) and monitored for at least 30 min [28]; MLV solubilisation (clearance) to give discoidal HDL particles decreases the absorbance at 325 nm. The presence of KBr prevents settling of the MLV during measurements. The time courses for MLV clearance were fitted by non-linear regression (two-phase exponential decay) using Prism (Graphpad Software), after normalising the data by adjusting the initial absorbance to 1 [27] and subtracting the background absorbance arising from glycation-induced AGE formation on apoA-I.

### Cell studies

J774A.1 murine macrophages (ATCC, TIB-67) were cultured and incubated with acetylated LDL (AcLDL, 200 µg apoB/ml, 24 h) as previously [9]. Cells were subsequently washed and incubated overnight in media containing BSA (0.2% w/v) and 8-(4-chlorophenylthio)adenosine 3′,5′-cyclic monohydrate phosphate (cAMP; 0.3 mM) [29]. For the drHDL experiments, cells were incubated ±5 µM 9-cis-retinoic acid and TO-901317 (N-(2,2,2-trifluoro-ethyl)-N-[4-(2,2,2-tri- fluoro- 1-hydroxy-1-trifluoromethyl-ethyl)-phenyl]- benzenesulfonamide; Sigma-Aldrich, St. Louis, USA) [13]. Cells were then washed and exposed to media containing BSA (0.2% w/v) for up to 8 h without or with 50 µg protein/ml apoA-I or drHDL to induce efflux. Media was collected as indicated, and the cells washed prior to lysis in water. Media and lysates were analysed for cholesterol and cholesteryl esters by HPLC [9]. Cell viability and number were determined by lactate dehydrogenase (LDH) release and protein concentrations respectively [9].

### Statistical Analysis

Data are mean ± SD from at least three independent experiments each with triplicate samples. Statistical analysis was performed by two-tailed t-test, or one-way or two-way ANOVA and Tukey's *post hoc* analysis; *p*<0.05 was taken as statistically significant.

## Results

### Characterisation of in vitro glycated lipid-free apoA-I and drHDL

Glucose (0–30 mM) did not induce significant Arg, Lys and Trp loss from either lipid-free apoA-I or drHDL (Table 2) consistent with insignificant levels of glycation and/or oxidation of these materials. In contrast, methylglyoxal and glycolaldehyde induced significant concentration-dependent losses. Arg loss was more extensive with methylglyoxal, whereas Lys and Trp loss was more marked with glycolaldehyde (Table 2). Glycolaldehyde induced CML formation on lipid-free apoA-I in a concentration-dependent manner (Table 2).

**Table 2:** Loss of Arg, Lys and Trp (% of controls) and CML formation (nmol/mg protein) on glycated lipid-free apoA-I and drHDL.

	Arg	Lys	Trp	CML
**Lipid-free apoA-I**				
Control	100±5	100±6	100±2	0.02±0.01
Glucose: 15 mM	106±7	95±4	107±15	ND
30 mM	90±4	87±2	86±2	ND
Methylglyoxal: 1.5 mM	67±16*	71±11*	76±11*	ND
3 mM	57±2*	69±2*	73±1*	ND
15 mM	46±7*	40±8*	44±9*	ND
30 mM	45±2*	41±1*	48±2*	ND
Glycolaldehyde: 0.3 mM	99±2	94±2	97±1	0.58±0.04^a^
1.5 mM	89±4*	73±8*	77±10*	8.61±0.40^b^
3 mM	93±3	76±1*	77±2*	16.33±0.06^c^
7.5 mM	99±1	56±2*	47±3*	16.98±4.53^c^
15 mM	88±8*	27±8*	19±5*	21.50±2.71^d^
30 mM	76±2*	13±3*	11±4*	34.72±0.84^e^
**drHDL**				
Control	100±8	100±1	100±1	ND
Glucose: 30 mM	101±1	96±3	95±5	ND
Methylglyoxal: 3 mM	59±1*	75±3*	86±3*	ND
30 mM	49±2*	51±3*	62±1*	ND
Glycolaldehyde: 3 mM	102±1	83±1*	96±3	ND
30 mM	97±2	18±2*	19±3*	ND

Data are expressed relative to control apoA-I (16 Arg, 21 Lys, 4 Trp).

* Significantly different to 0 mM (one-way ANOVA). Statistical differences for CML data (one-way ANOVA):

a versus control;

b versus control and 0.3 mM glycolaldehyde;

c versus control, 0.3 and 1.5 mM glycolaldehyde;

d versus control, 0.3, 1.5 and 3 mM glycolaldehyde;

e versus control, 0.3,1.5, 3, 7.5 and 15 mM glycolaldehyde.

ND, not determined.

**Figure 1:**
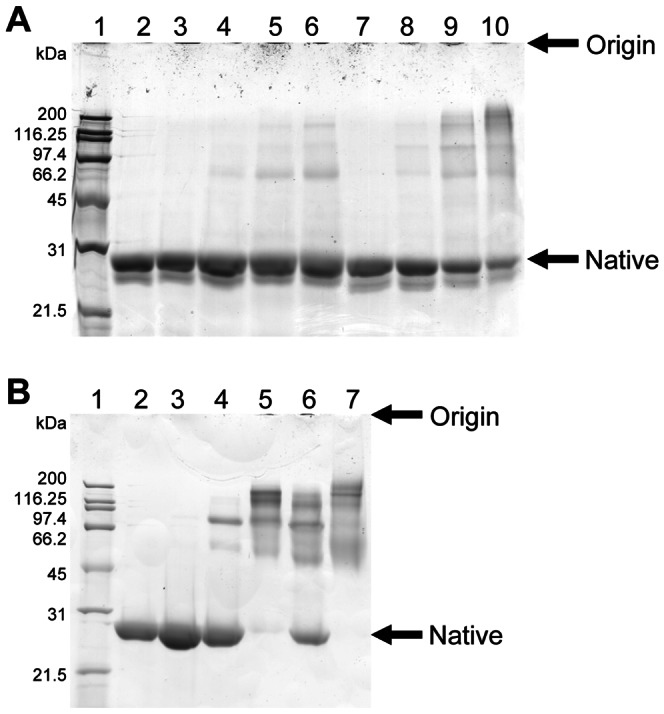
Cross-linking of lipid-free apoA-I and drHDL induced by glucose and reactive aldehydes. SDS-PAGE of (A) lipid-free apoA-I or (B) drHDL after exposure to glucose, methylglyoxal or glycolaldehyde for 24 h at 37°C. For both gels: lane 1, molecular mass markers (kDa); lane 2, control lipid-free apoA-I or drHDL; lane 3, apoA-I or drHDL modified by 30 mM glucose. (A) Lanes 4–10: apoA-I modified by 0.3 mM methylglyoxal (lane 4), 1.5 mM methylglyoxal (lane 5), 3 mM methylglyoxal (lane 6), 0.03 mM glycolaldehyde (lane 7), 0.3 mM glycolaldehyde (lane 8), 1.5 mM glycolaldehyde (lane 9), or 3 mM glycolaldehyde (lane 10). (B) Lanes 4–7: drHDL modified by 3 mM methylglyoxal (lane 4), 30 mM methylglyoxal (lane 5), 3 mM glycolaldehyde (lane 6) or 30 mM glycolaldehyde (lane 7). Representative gel of three.

Methylglyoxal- and glycolaldehyde-, but not glucose-, induced significant cross-linking of lipid-free apoA-I and apoA-I in drHDL (Fig. 1). A greater degree of crosslinking was detected with glycolaldehyde-modified lipid-free apoA-I than methylglyoxal apoA-I for the same concentration of aldehyde (e.g. lane 6 versus lane 10, Fig. 1A). drHDL composition or particle size were not affected by glycolaldehyde (data not shown). Methylglyoxal did not alter drHDL composition, but induced a small decrease in particle diameter (9.7 to 9.0 nm) at high concentrations [15].

### Clearance of phospholipid multilamellar vesicles (MLV) by control and glycated apoA-I

**Figure 2:**
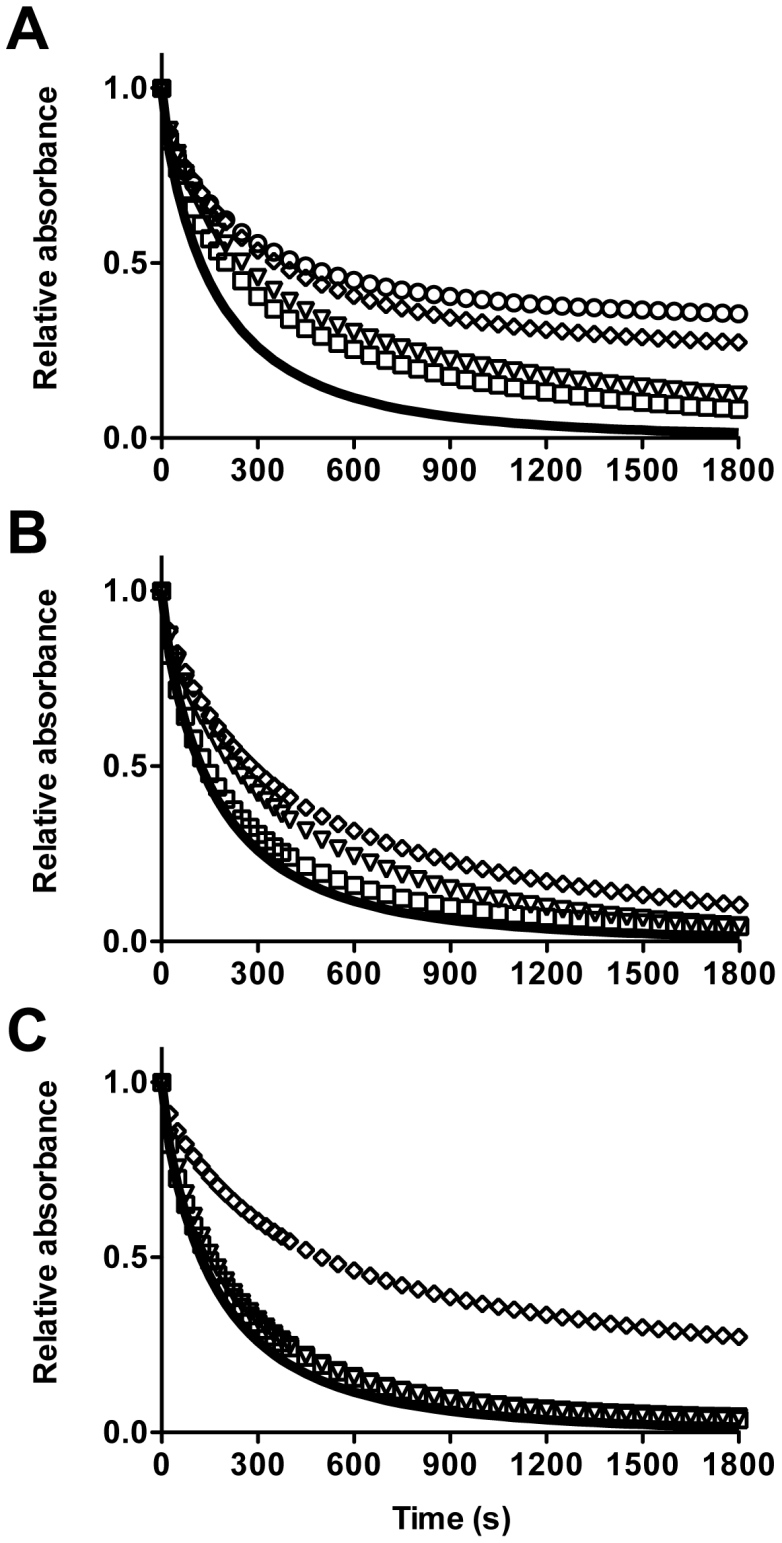
Clearance of DMPC multilamellar vesicles over time by native and glycated lipid-free apoA-I. ApoA-I was pretreated with 0–30 mM glucose (A), 0–3 mM methylglyoxal (B) or 0–3 mM glycolaldehyde (C) for 24 h at 37°C, before mixing with DMPC MLV and monitored at 325 nm. Solid line, control apoA-I (0 mM glucose/methylglyoxal/glycolaldehyde); squares, 5.5 mM glucose or 0.03 mM aldehyde; triangles, 10 mM glucose or 0.03 mM aldehyde; diamonds, 20 mM glucose or 3 mM aldehyde; circles 30 mM glucose. Lines are plotted from the mean absorbance values obtained from triplicate samples in a representative experiment. Error bars are omitted for clarity.

**Figure 3:**
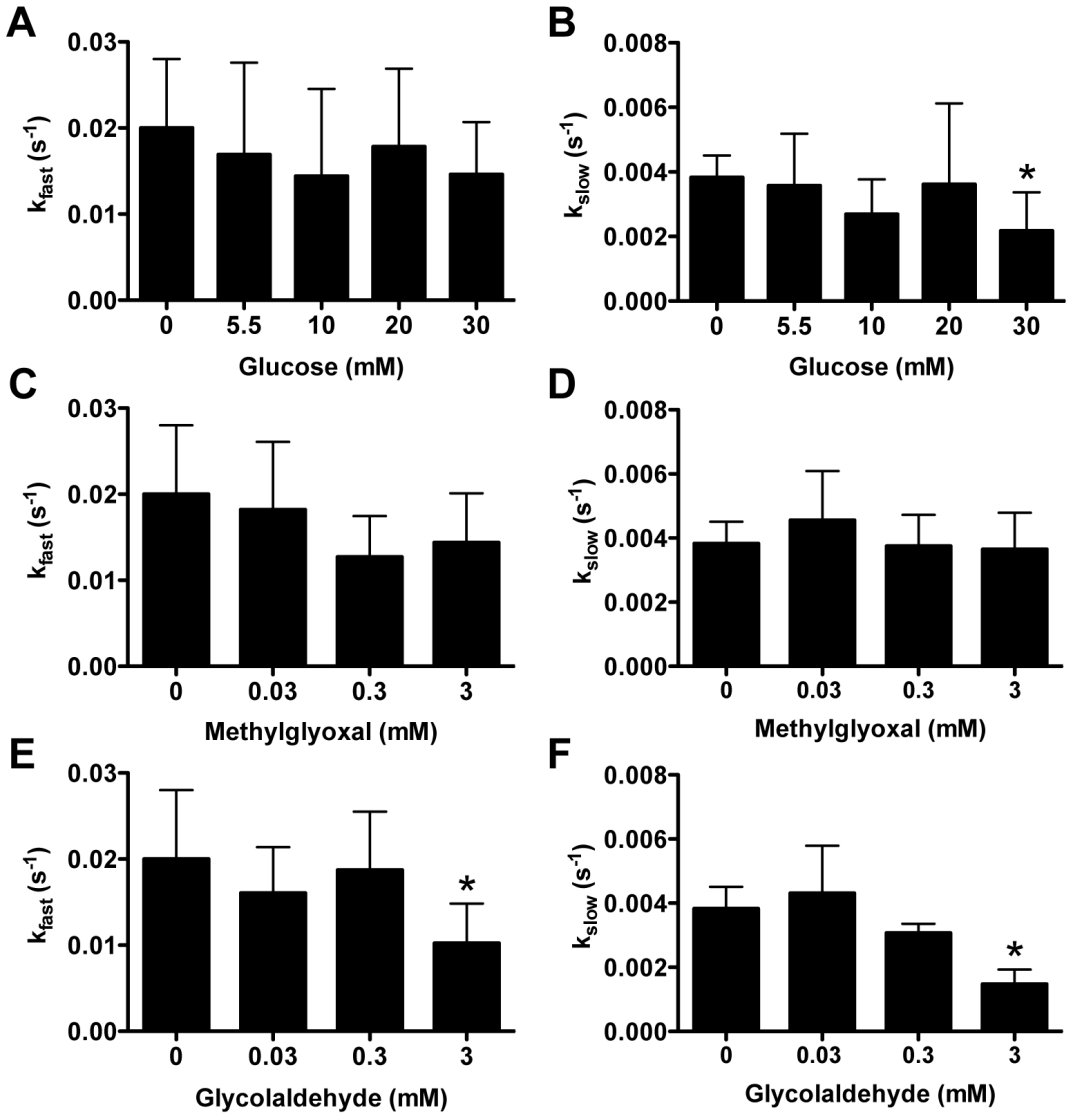
Kinetic parameters of DMPC multilamellar vesicle clearance by glycated lipid-free apoA-I. Two-phase exponential decay equations were fitted to glucose (A, B), methylglyoxal (C, E) and glycolaldehyde-modified apoA-I (D, F) time course clearances of DMPC MLV to determine fast (A, C, E) and slow rate constants (B, D, F) * Significantly different by repeated measures one-way ANOVA to the complete system without apoA-I pretreatment with glucose/methylglyoxal/glycolaldehyde.

Pretreatment of lipid-free apoA-I with glucose (Fig. 2A), methylglyoxal (Fig. 2B), or glycolaldehyde (Fig. 2 C) reduced the rate of DMPC MLV clearance with the change in rate dependent on the concentration of the modifying agent. Analysis using a two-phase exponential decay [27], allowed fast and slow rate constants to be determined. The rate constant for the slower of the two processes, k_slow_ was significantly reduced on pretreatment with 30 mM glucose (Fig. 3 B), however neither k_fast_ or k_slow_ were affected by methylglyoxal-modified lipid-free apoA-I at the concentrations of methylglyoxal used (0–3 mM; Fig. 3C, D). Significant inhibition of DMPC MLV clearance was however detected when 30 mM methylglyoxal was used as a positive control (data not shown). k_fast_ and k_slow_ were significantly decreased by 3 mM glycolaldehyde-modified lipid-free apoA-I (Fig. 3E, F) compared to control apoA-I.

### Macrophage cholesterol efflux to glycated versus control lipid-free apo A-I

**Figure 4:**
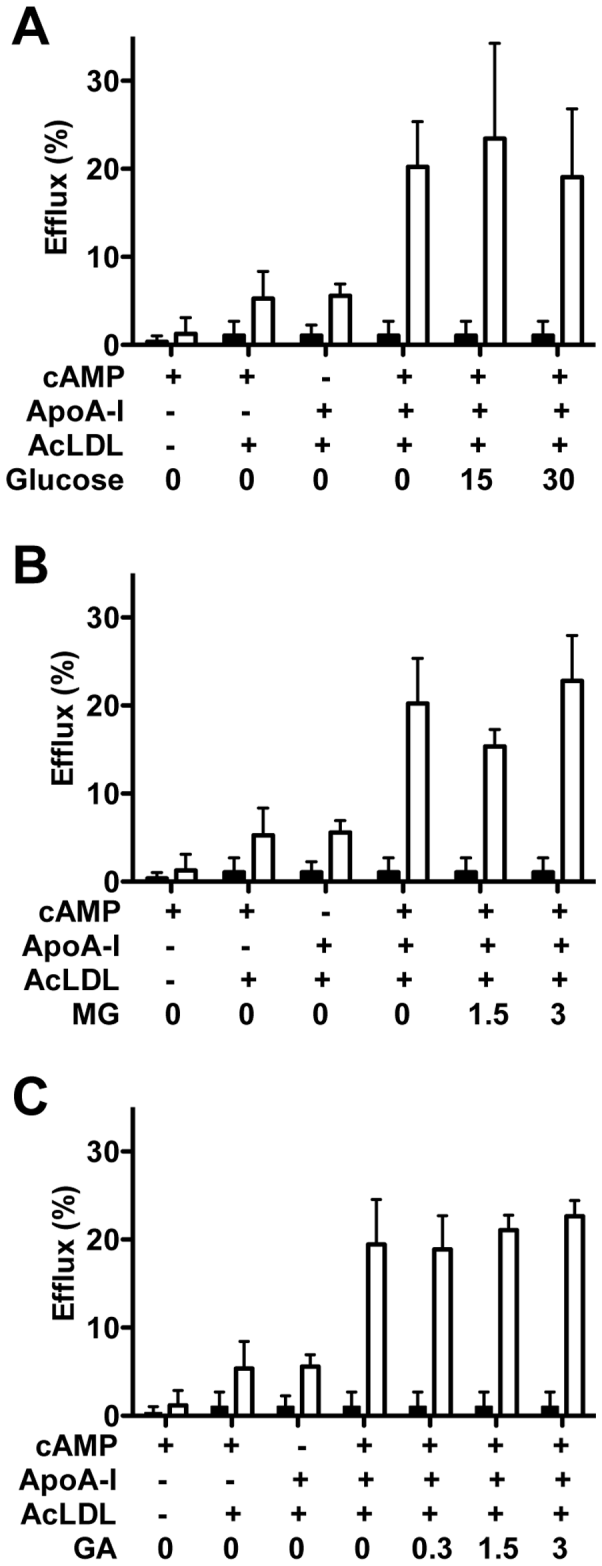
Cholesterol efflux to native and glycated lipid-free apoA-I from lipid-laden macrophages. AcLDL-loaded J774A.1 cells were pretreated with cAMP, before exposure to control or modified apoA-I for 0 (black bars) or 4 h (white bars). Lipid free apoA-I was treated with (A) 0–30 mM glucose, (B) 0–3 mM methylglyoxal (MG), or (C) 0–3 mM glycolaldehyde (GA) for 24 h at 37°C before addition to cells. * Significantly different by two-way ANOVA to the complete system without apoA-I pretreatment with glucose/methylglyoxal/glycolaldehyde at that time point.

Exposure of J774A.1 murine macrophages to AcLDL increased cellular total cholesterol relative to controls (38±12 versus 144±28 nmol cholesterol/mg cell protein) resulting in the formation of model lipid-laden cells. Exposure to lipid-free apoA-I (50 µg/ml; within previous concentration ranges [20]–[22], [30]) resulted in lipid efflux; this was stimulated approximately 4-fold by treatment with a cAMP derivative (Fig. 4A). The amount of cholesterol detected in the media after this treatment was 32±10 nmoles/mg cell protein. This treatment did not affect cell viability or protein levels (data not shown). Efflux reached a plateau after 4 h (data not shown). Efflux from the cAMP derivative-stimulated lipid-laden cells to apoA-I was not significantly affected by pre-glycation of the protein with 15–30 mM glucose (Fig. 4A), 1.5 or 3 mM methylglyoxal (Fig. 4B), or 0.3, 1.5 or 3 mM glycolaldehyde (Fig. 4C). Efflux was however decreased by >50% to apoA-I modified by higher levels (15 or 30 mM) glycolaldehyde used as a positive control (from 32±10 to 15±9 nmoles/mg cell protein for 15 mM glycolaldehyde or 9±2 nmoles/mg cell protein for 30 mM glycolaldehyde; data not shown).

### Macrophage cholesterol efflux to drHDL containing glycated or unmodified apoA-I

**Figure 5:**
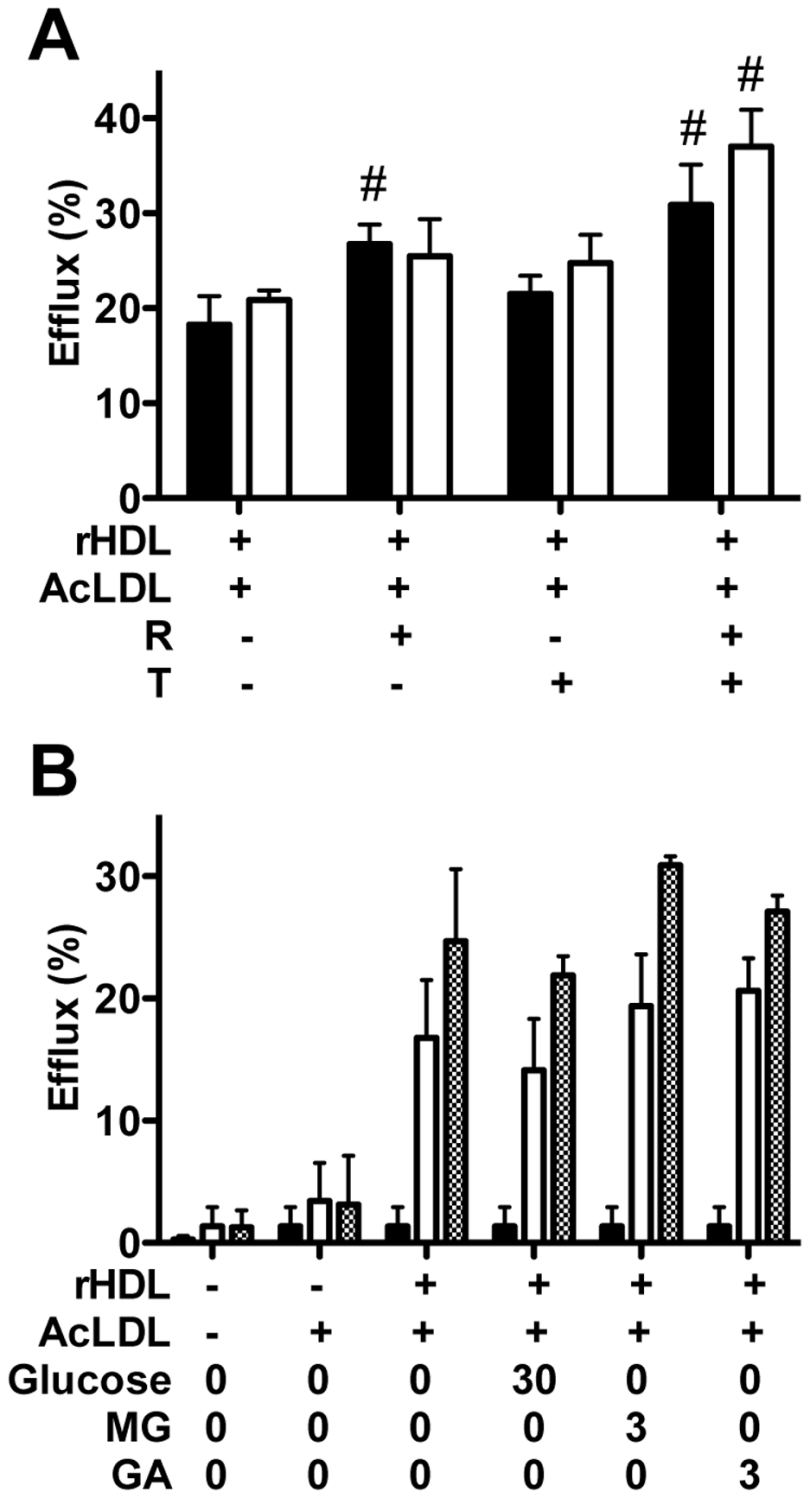
Cholesterol efflux to native and glycated drHDL from lipid-laden mouse macrophages. (A) Cholesterol efflux from AcLDL-loaded J774A.1 cells exposed to 5 µM 9-cis-retinoic acid (R) and/or TO-901317 (T) after exposure (8 h) to control drHDL (black bars) or drHDL exposed to glycolaldehyde (30 mM, 24 h, white bars). # Significantly different to control as assessed by one-way ANOVA. (B) Macrophage cholesterol efflux from AcLDL-loaded J774A.1 cells, following pretreatment with 5 µM 9-cis-retinoic acid (R) and TO-901317 (T), to drHDL containing apoA-I after 0 (black bars), 4 (white bars) or 8 h (dotted bars). drHDL was treated with 0–30 mM glucose, 3 mM methylglyoxal (MG) or 3 mM glycolaldehyde (GA) for 24 h, 37°C.

Cholesterol efflux to native drHDL was unaffected by cAMP treatment (not shown), but was increased by 9-cis-retinoic acid, either alone or with TO-901317 (Fig. 5A). Glycation of apoA-I in drHDL with 30 mM glucose, 3 mM methylglyoxal or 3 mM glycolaldehyde (Fig. 5B) did not affect efflux, irrespective of pre-treatment with cAMP (data not shown) or LXR-RXR agonists (Fig. 5A). Efflux to drHDL significantly increased between 4 and 8 h (Fig. 4B) irrespective of protein glycation or not.

### Inhibition of in vitro apoA-I glycation and restoration of efflux

**Figure 6:**
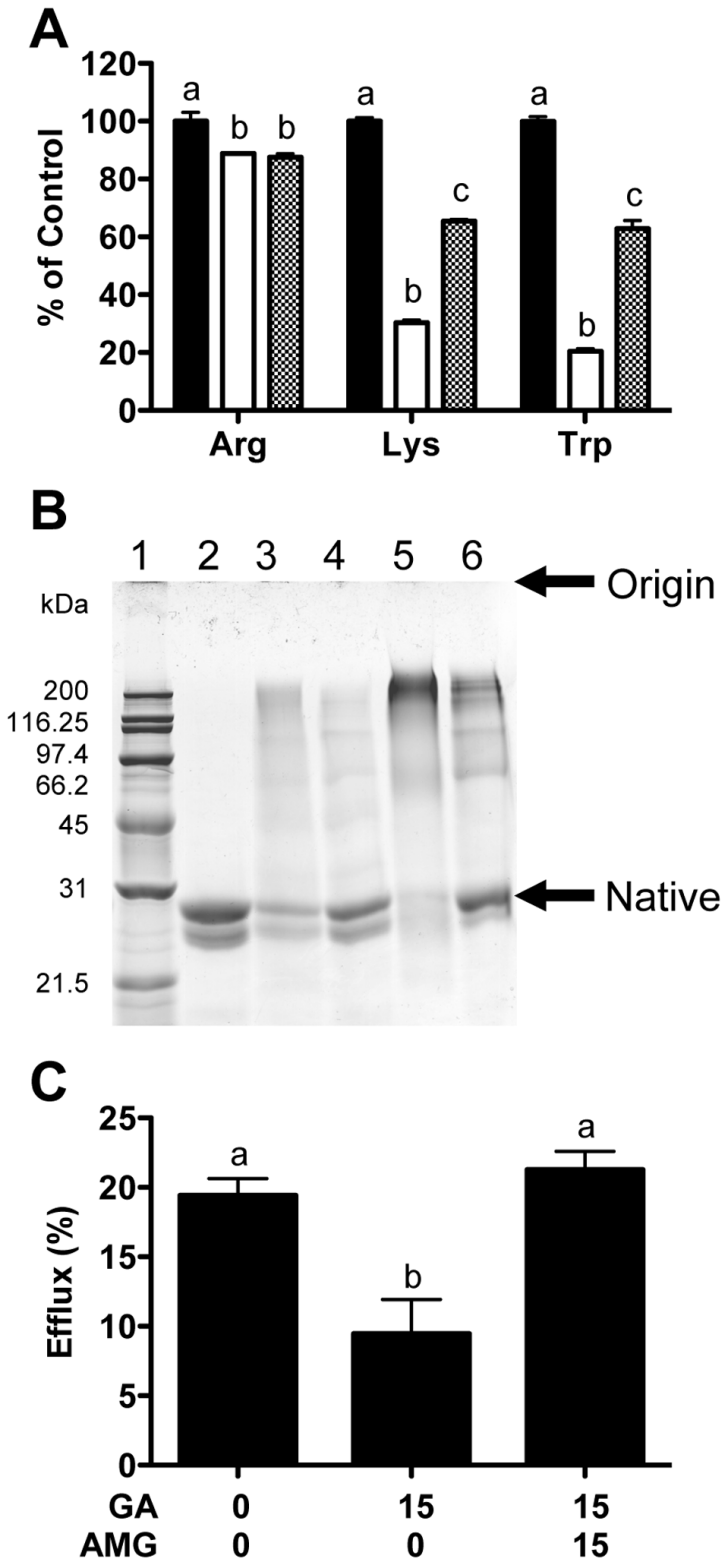
Inhibition of glycation of lipid-free apoA-I by aminoguanidine. (A) Arg, Lys and Trp loss in lipid-free apoA-I exposed to 0 (black bars) or 15 mM glycolaldehyde in the absence (white bars) or presence (spotted bars) of 15 mM aminoguanidine (24 h, 37°C). (B) SDS-PAGE of lipid-free apoA-I exposed to 0 mM glycolaldehyde (lane 2), 3 mM glycolaldehyde (lane 3), 3 mM glycolaldehyde and 3 mM aminoguanidine (lane 4), 15 mM glycolaldehyde (lane 5), 15 mM glycolaldehyde and 15 mM aminoguanidine (lane 6) for 24 h. Lane 1: molecular mass markers. Representative gel of 3. (C) Cholesterol efflux after 4 h to lipid-free apoA-I exposed (24 h, 37°C) to 0 or 15 mM glycolaldehyde (GA) ± aminoguanidine (AMG, 15 mM). Columns with different superscript letters are significantly different (one-way ANOVA).

Aminoguanidine (15 mM) present during the *in vitro* glycation of lipid-free apoA-I with glycolaldehyde (15 mM) decreased the extent of loss of Lys and Trp residues, but did not affect the loss of Arg residues (Fig. 6A). Equimolar addition of aminoguandine (3 or 15 mM) to glycolaldehyde and apoA-I incubations also inhibited crosslink formation (lane 3 versus 4, and lane 5 versus 6, Fig. 6B.) This treatment restored efflux to glycated lipid-free apoA-I to control apoA-I levels (Fig. 6C).

### Characterisation of in vivo modified apoA-I and cholesterol efflux to lipid-free apoA-I from people with Type 1 diabetes and controls

**Figure 7:**
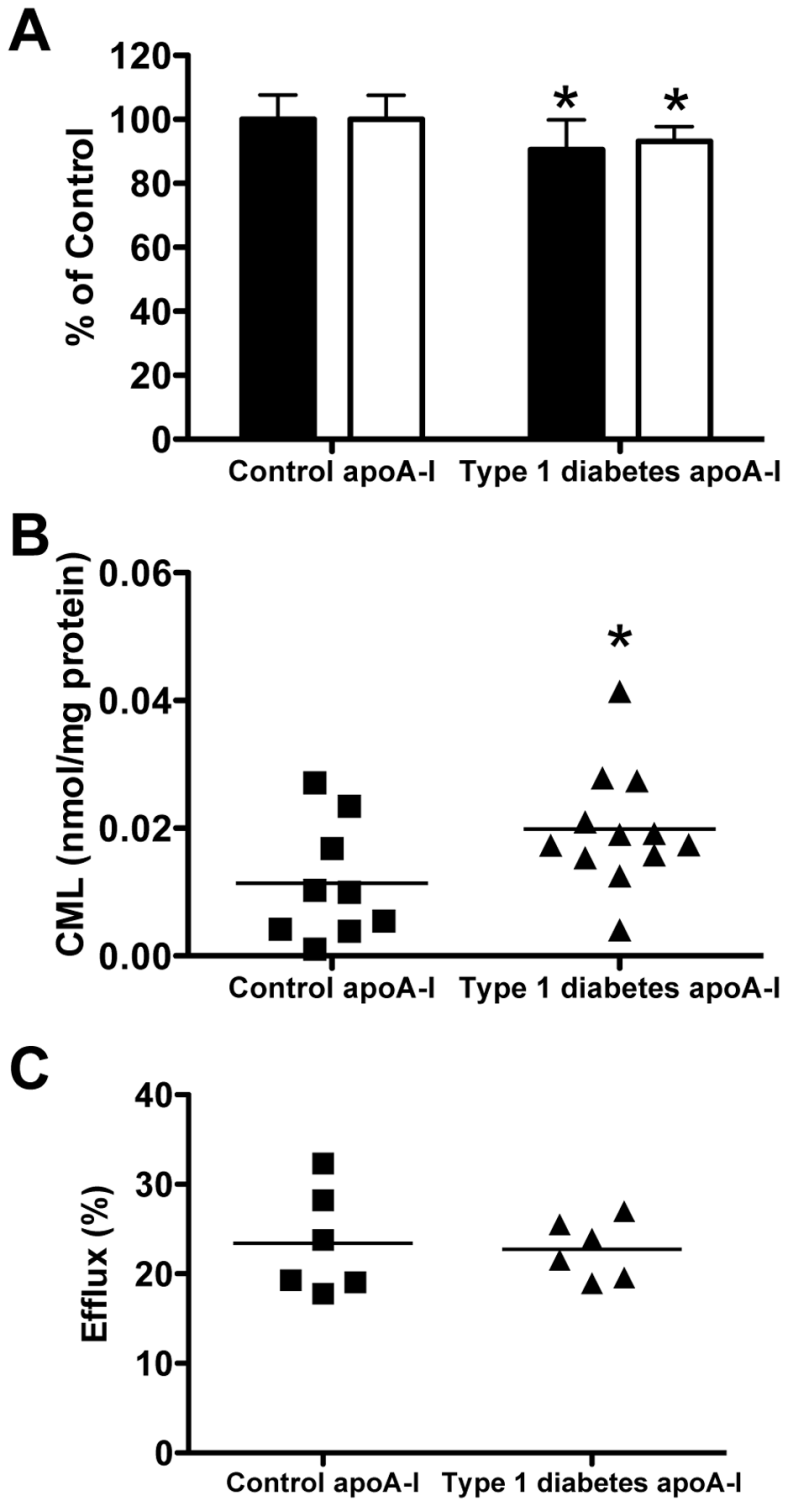
Comparison of lipid-free apoA-I isolated from people with Type 1 diabetes with controls. (A) Loss of Arg (black bars) and Lys (white bars), (B) CML concentrations and (C) efflux of cholesterol from lipid-laden macrophages over 4 h. * Significantly different to control as determined by two-tailed t-tests.

ApoA-I from people with well-controlled Type 1 diabetes had lower Arg and Lys than controls (Arg: 90.5±9.4% vs 100.0±7.6%; Lys: 93.2±4.5% vs 100.0±7.6; both *p*<0.05) (Fig. 7A). Trp levels were not different, but CML levels were elevated (1.75-fold; Fig. 7B). No cross-linked apoA-I was detected in patients or controls (data not shown). Efflux (at 4 h) from lipid-laden macrophages to lipid-free apoA-I from people with diabetes, or controls, was not significantly different (Fig. 7C).

## Discussion

Cholesterol efflux from lipid-laden macrophages to lipid-free apoA-I or HDL is part of the anti-atherogenic reverse cholesterol transport pathway [12]. Hyperglycaemia-induced changes to these lipoproteins may enhance atherosclerosis [31]. Previous studies on the effects of glycation on cholesterol efflux have yielded mixed data [21]–[23], [30], [32] with this potentially reflecting the poorly-characterised nature/extent of particle modification, heterogeneous HDL populations, different cell types and whether the cells examined were lipid-loaded or not. We have attempted to elucidate the factors that modulate phospholipid association with apoA-I, and cholesterol efflux by employing well-characterised lipid-free apoA-I, and drHDL particles containing apoA-I as the sole protein. These materials were produced with controlled and defined levels of glycation, and insignificant levels of oxidation, factors that have not been addressed in detail in previous studies. As the glycation protocol employed does not result in significant protein or lipid oxidation a role for oxidation in the observed changes can be discounted [15].

Glucose did not modify lipid-free apoA-I or drHDL significantly, whereas methylglyoxal and glycolaldehyde induced rapid modification, consistent with previous studies [14], [15]. Greater loss of Lys and Trp was detected with glycolaldehyde, compared to methylglyoxal, with both lipid-free apoA-I and drHDL. Methylglyoxal induced a similar loss of each residue for lipid-free apoA-I, and a preferential loss of Arg from drHDL [25]. This was accompanied by protein cross-linking. ApoA-I from people with Type 1 diabetes showed significant Arg and Lys depletion, but not Trp loss compared to controls, consistent with the known kinetics of modification of side-chain residues by these agents [33]. This *in vivo* loss was greater than that observed for apoA-I exposed to glucose *ex vivo*, but less than that induced by methylglyoxal or glycolaldehyde. Previous studies have reported no differences between HDL from controls or people with Type 1 diabetes with regard to size, density and particle composition [34].

Exposure of isolated apoA-I to glycolaldehyde *ex vivo* increased CML levels; elevated levels were also detected on apoA-I isolated from people with Type 1 diabetes compared to controls. We have previously reported increased levels of CML and others AGEs on lipid-free apoA-I isolated from people with Type 2 diabetes [14]. Ten-fold higher levels of CML have also been reported on HDL isolated from people with diabetes and secondary kidney disease when compared to controls [22]. These data suggest that AGE formation on apoA-I *in vivo* arises primarily from the reactions of reactive aldehydes as the plasma lifetime of apoA-I and HDL is short [35].

Glycation of apo-A-I by 30 mM glucose or 3 mM glycolaldehyde impaired the clearance of DMPC MLV compared to control apoA-I. Analysis of the kinetic data using a two-phase exponential decay [27], show that both the fast and slow components of the reaction are affected by 3 mM glycolaldehyde, and only the slow component for 30 mM glucose. Previous studies have examined the rate of clearance (solubilisation) of DMPC MLV and shown that it is affected by apoE isoforms and fragments [27], various apoA-IV N-terminus mutants [36], and glycation of apoA-I by fructose or artificial sweetners [16]. Other studies have reported that the affinity of antibodies for the lipid-binding region of apoA-I is altered by methylglyoxal exposure [14], [15]. These previous studies, together with the current data, suggest that the structural changes to apoA-I induced by glucose or glycolaldehyde occur, at least in part, in the lipid-binding regions of the protein, with this resulting in reduced phospholipid-protein interactions, and decreased conversion of lipid-free apoA-I to discoidal HDL particles. These effects however only occur to a significant extent with high levels of glucose and reactive aldehydes.

As interaction of apoA-I with phospholipids is a requirement for ABCA1-mediated cholesterol efflux and formation of discoidal HDL [11], [12], we examined the effects of different extents of glycation on macrophage cholesterol efflux to control and modified apoA-I particles. Cholesterol efflux was unchanged to lipid-free apoA-I modified by 30 mM glucose, 3 mM methylglyoxal, or 3 mM glycolaldehyde from J774A.1 cells. Consistent with this data a recent study has reported that modification of apoA-I with low concentrations of methylglyoxal and glycolaldehyde (250 µM) did not affect cholesterol efflux from human ABCA1-expressing baby hamster kidney cells [32]. These data contrast with the reported impaired efflux from (non-lipid-loaded) macrophages to lipid-free apoA-I modified with 500 mM glucose for ≥4 weeks [22]. This extensive, high concentration exposure protocol would be expected to induce major protein changes including oxidation (which was not assessed), which may rationalise these divergent results. Furthermore this exposure time and glucose concentration are unlikely to be biologically relevant given the short plasma half-life of apoA-I [35] and the maximum levels of glucose detected in people with poorly-controlled diabetes (<30 mM) [7]. This group also reported decreased efflux from non-lipid-loaded THP-1 cells to lipid-free apoA-I modified by ≥1 mM methylglyoxal, and AGE-HDL prepared by incubating HDL with 500 mM ribose [22]. These results suggest that human ABCA1 may be more sensitive to glycated lipid-free apoA-I than mouse ABCA1.

The extent of cholesterol efflux from lipid-laden cells to lipid-free apoA-I isolated from people with complication-free Type 1 diabetes, and healthy subjects, did not differ consistent with the low levels of protein modification detected. Whether this is also true for apoA-I from people with poorly-controlled diabetes, or severe complications (e.g. renal failure), where protein modification may be greater [22], remains to be established.

Efflux to drHDL was also unchanged regardless of the modifying agent. Efflux to discoidal or spherical HDL occurs predominantly via ABCG1-dependent pathways [12], [13], unlike the lipid-free apoA-I ABCA1-dependent pathway. Matsuki *et al* [23] have reported decreased efflux from non-loaded THP-1 cells to human HDL modified by 100 mM 3-deoxyglucosone (a level not achieved *in vivo*) for 7 days even in the presence of increased ABCG1 mRNA and protein expression. Extensive modification induced by this treatment, together with possible oxidation and heterogeneity of the HDL used, may explain these differences. Efflux via SR-BI [11] does not appear to be modulated, as efflux to (phospholipid-containing) drHDL was unchanged by glycation.

Use of lipid-free apoA-I modified with higher concentrations of glycolaldehyde (15 mM) indicated that macrophage cholesterol efflux can be markedly reduced (by >50% compared to control apoA-I) with more extensive modification of the apoA-I. ApoA-I modification by 3 or 15 mM glycolaldehyde was partly inhibited by equimolar aminoguanidine, with this being sufficient to restore efflux to levels observed with control lipid-free apoA-I. Although aminoguanidine is unusable clinically [37], other anti-glycation agents which react rapidly with (and hence remove) reactive aldehdyes [38]–[40] may merit further study. Hydralazine, which inhibits glycation [40], decreases AGE formation in a Type 2 diabetes model, and improves renal function [41].

Although the aldehyde concentrations employed here are higher than those reported in plasma (≤0.5 mM [7]), the latter represent steady-state (i.e. residual material that has not reacted with plasma components), rather than absolute concentrations to which proteins are likely to be exposed over their biological lifetime. Methylglyoxal concentrations in cells and tissues, such as within the artery wall, may be significantly greater than this as a result of formation of this material intracellularly via increased triosephosphate formation (glycolytic metabolism, the Embden-Meyerhof pathway) and subsequent degradation [6]. Thus methylglyoxal levels have been reported to be 20-fold high in the lens than in plasma [42]. Protein modification *in vivo* occurs over extended periods via continual exposure to these sub-millimolar levels of methylglyoxal, and the modifications induced by such exposure are likely to accumulate over time. At present it is unclear how such continual exposure compares to bolus treatment, as employed here. However it has been reported that methylglyoxal has a plasma lifetime of seconds - minutes (the rate constant for initial reaction of methylglyoxal with *N*-acetylarginine is reported as 8.5×10^−3^ M^−1^ s^−1^ in [33], yielding a half-life, t_1/2_, of approximately 80 s) and apoA-I has a lifetime of 24 h (or greater at sites where it may be retained) and therefore the total flux of methylglyoxal to which this protein will be exposed is likely to be orders of magnitude greater than the plasma steady-state level described above. CML levels detected in this study with 3 mM glycolaldehyde (approximately 16 nmoles/mg apoA-I, 7 µg CML/mg), lie within the range reported by others for HDL of people with diabetes and renal deficiency [22], also suggesting that the damage induced by these bolus concentrations may be pathologically relevant.

Overall, these data indicate that apoA-I glycation, using relatively modest excesses of glucose and reactive aldehydes can inhibit phospholipid association, but not macrophage cholesterol efflux. Modulation of these processes requires significant protein modification, and may arise from conformational or amino acid side-chain modifications within the lipid-binding regions of apoA-I. These changes are more extensive than those detected on apoA-I from people with complication-free Type 1 diabetes, but poor glycaemic control, and severe disease, may result in a greater extent of protein modification such that this impairment of efflux could be of relevance. Glycation inhibitors can attenuate such apoA-I modification and prevent impaired efflux, suggesting that such compounds may benefit people with diabetes with impaired reverse cholesterol transport.
